# Connexin-Mediated Signaling in Nonsensory Cells Is Crucial for the Development of Sensory Inner Hair Cells in the Mouse Cochlea

**DOI:** 10.1523/JNEUROSCI.2251-16.2016

**Published:** 2017-01-11

**Authors:** Stuart L. Johnson, Federico Ceriani, Oliver Houston, Roman Polishchuk, Elena Polishchuk, Giulia Crispino, Veronica Zorzi, Fabio Mammano, Walter Marcotti

**Affiliations:** ^1^Department of Biomedical Science, University of Sheffield, S10 2TN Sheffield, United Kingdom,; ^2^Department of Physics and Astronomy “G. Galilei,” University of Padua, 35131 Padua, Italy,; ^3^Venetian Institute of Molecular Medicine, Foundation for Advanced Biomedical Research, 35129 Padua, Italy,; ^4^Department of Biomedical Sciences, Institute of Cell Biology and Neurobiology, Italian National Research Council, 00015 Monterotondo, Italy, and; ^5^Telethon Institute of Genetics and Medicine, Pozzuoli, 80131 Napoli, Italy

**Keywords:** cochlea, connexin, deafness, development, gap-junction, inner hair cells

## Abstract

Mutations in the genes encoding for gap junction proteins connexin 26 (Cx26) and connexin 30 (Cx30) have been linked to syndromic and nonsyndromic hearing loss in mice and humans. The release of ATP from connexin hemichannels in cochlear nonsensory cells has been proposed to be the main trigger for action potential activity in immature sensory inner hair cells (IHCs), which is crucial for the refinement of the developing auditory circuitry. Using connexin knock-out mice, we show that IHCs fire spontaneous action potentials even in the absence of ATP-dependent intercellular Ca^2+^ signaling in the nonsensory cells. However, this signaling from nonsensory cells was able to increase the intrinsic IHC firing frequency. We also found that connexin expression is key to IHC functional maturation. In Cx26 conditional knock-out mice (*Cx26^Sox10-Cre^*), the maturation of IHCs, which normally occurs at approximately postnatal day 12, was partially prevented. Although Cx30 has been shown not to be required for hearing in young adult mice, IHCs from Cx30 knock-out mice exhibited a comprehensive brake in their development, such that their basolateral membrane currents and synaptic machinery retain a prehearing phenotype. We propose that IHC functional differentiation into mature sensory receptors is initiated in the prehearing cochlea provided that the expression of either connexin reaches a threshold level. As such, connexins regulate one of the most crucial functional refinements in the mammalian cochlea, the disruption of which contributes to the deafness phenotype observed in mice and DFNB1 patients.

**SIGNIFICANCE STATEMENT** The correct development and function of the mammalian cochlea relies not only on the sensory hair cells, but also on the surrounding nonsensory cells. Although the nonsensory cells have been largely implicated in the general homeostasis in the mature cochlea, their involvement in the initial functional differentiation of the sensory inner hair cells is less clear. Using mutant mouse models for the most common form of congenital deafness in humans, which are knock-outs for the gap-junction channels connexin 26 and connexin 30 genes, we show that defects in nonsensory cells prevented the functional maturation of inner hair cells. In connexin knock-outs, inner hair cells remained stuck at a prehearing stage of development and, as such, are unable to process sound information.

## Introduction

In mammals, the sense of hearing relies on mechanoelectrical transduction performed by the primary sensory receptor inner hair cells (IHCs) and the outer hair cells (OHCs). Functionally mature IHCs relay sound information to Type I spiral ganglion neurons with high temporal precision via the graded release of glutamate from their ribbon synapses (Fuchs, 2005). Before the onset of hearing, which in most rodents occurs at approximately postnatal day 12, spontaneous Ca^2+^ action potential (AP) activity in IHCs plays a role in driving the refinement of the immature auditory circuitry (Johnson et al., 2011, 2013; Clause et al., 2014). These sensory hair cells are embedded in a matrix of epithelial nonsensory cells, which are crucial for normal cochlear function (Monzack and Cunningham, 2013). Nonsensory cells in the mammalian cochlea are interconnected by a network of gap junction channels, which are intercellular conduits formed by the head-to-head docking of two hemichannels from adjacent cells (Goodenough and Paul, 2009) creating an extensive functional syncytium in the cochlear sensory epithelium (Kikuchi et al., 1995). Gap junction channels in the mammalian cochlea are primarily formed by Cx26 and Cx30 (Ahmad et al., 2003) and mutations of the genes encoding for these two proteins (*Gjb2* and *Gjb6*, respectively) are associated with the most common form of prelingual congenital hearing impairment in humans (DFNB1) (Xu and Nicholson, 2013). Classically, the connexin-based gap junction network in the adult cochlea is thought to contribute to cochlear homeostasis (Zdebik et al., 2009). Mouse models confirmed that Cx26 and Cx30 are involved in a wide range of activities important for the normal function of the developing and mature hearing system (Mammano, 2013; Jagger and Forge, 2015; Kelly et al., 2015; Wingard and Zhao, 2015; Zhu et al., 2015).

Connexin hemichannels are known to mediate paracrine signaling by opening in response to a rise in the cytosolic free Ca^2+^ concentration ([Ca^2+^]_c_) (Leybaert et al., 2003; De Vuyst et al., 2006). The opening of hemichannels releases intracellular ATP into the extracellular milieu, which promotes signal encoding by [Ca^2+^]_c_ oscillations (Uhlen and Fritz, 2010; Parekh, 2011) and conveys crucial biochemical information throughout the cochlear sensory epithelium via intercellular Ca^2+^ waves (Leybaert and Sanderson, 2012; Mammano, 2013). In the immature mouse cochlea, ATP-induced signaling from connexin extrajunctional hemichannels (Anselmi et al., 2008; Majumder et al., 2010) has been proposed to induce Ca^2+^-dependent AP activity in sensory IHCs both directly (Tritsch et al., 2007) and indirectly (Wang et al., 2015), although a more modulatory role for ATP on IHC firing has also been shown (Johnson et al., 2011). Despite the involvement of Cx26 and Cx30 in cochlear function and hearing, their exact role and the relative contribution of the two connexins to mammalian cochlear physiology during prehearing stages of development are still unclear (Liu et al., 2016).

In this study, we used three different mouse models of connexin deficiency to provide evidence for a direct role of connexins in the functional maturation of prehearing mouse IHCs. Our results indicate that ATP-induced signaling in cochlear nonsensory cells is not required for generating APs in prehearing IHCs. However, this ATP-dependent signaling increases the frequency of the intrinsically generated AP activity in IHCs. We also found that a threshold level for the expression of both Cx26 and Cx30 is required for the acquisition of the mature sensory profile in IHCs.

## Materials and Methods

### 

#### 

##### Ethics statement.

In the United Kingdom, experiments were performed in accordance with Home Office regulations under the Animals (Scientific Procedures Act) 1986 and following approval by the University of Sheffield Ethical Review Committee. In Italy, animal work was approved by the Ethics committee of the University of Padua (Protocol 104230, October 12, 2013).

##### Tissue preparation.

Apical coil IHCs from transgenic mice of either *sex (Cx30(*−/−*)* (MGI:2447863): Teubner et al., 2003; *Cx30*^Δ/Δ^ (MGI:5486677): Boulay et al., 2013; *Cx26^Sox10Cre^*: Anselmi et al., 2008; Crispino et al., 2011) and their littermate controls were studied in acutely dissected organs of Corti from postnatal day 3 (P3) to P25, where the day of birth is P0. Targeted ablation of Cx26 in the nonsensory cells was achieved by crossing *Cx26^loxP/loxP^* (MGI:2183509) (Cohen-Salmon et al., 2002) mice with *Sox10-Cre* mice (MGI:3586900) (Matsuoka et al., 2005). Sox10 is predominantly expressed in glial cells of the nervous system (Kuhlbrodt et al., 1998), and in the cochlea it is found in the nonsensory cells of the greater epithelial ridge (GER, also known as Kölliker's organ) and in other supporting cells of the organ of Corti surrounding the IHCs and OHCs, but not in IHCs or OHCs (Watanabe et al., 2000). Genotyping protocols were performed by PCR using the primers previously described (Anselmi et al., 2008; Boulay et al., 2013). After killing the animals by cervical dislocation, cochleae were rapidly dissected (Marcotti et al., 2003) and kept in the following extracellular solution (in mm): 135 NaCl, 5.8 KCl, 1.3 CaCl_2_, 0.9 MgCl_2_, 0.7 NaH_2_PO_4_, 5.6 d-glucose, 10 HEPES-NaOH, 2 sodium pyruvate; MEM amino acids solution (50×, without l-glutamine) and MEM vitamins solution (100×) were added from concentrates (Fisher Scientific); pH was adjusted to 7.5, ∼308 mOsmol kg^−1^. Dissected cochleae were transferred to a microscope chamber, immobilized using a nylon mesh fixed to a stainless steel ring, and continuously perfused with the above extracellular solution. The sensory epithelia were viewed using an upright microscope (Leica, Olympus) with Nomarski differential interference contrast optics (63× water-immersion objectives and 10× or 15× eyepieces). All recordings were performed near body temperature (34°C–37°C) unless otherwise stated.

##### Whole-cell patch clamp.

Voltage and current recordings were performed using Axopatch 200B (Molecular Devices), EPC7 (HEKA), and Optopatch (Cairn Research) amplifiers. Patch pipettes, with resistances of 2–4 mΩ, were pulled from soda glass capillaries, and the shank of the electrode was coated with surf wax (Mr Zoggs Sex Wax). For current and voltage recordings, the pipette intracellular solution contained the following (in mm): 131 KCl, 3 MgCl_2_, 1 EGTA-KOH, 5 Na_2_ATP, 5 HEPES-KOH, 10 sodium phosphocreatine, pH 7.3; for cell-attached recordings, the pipette contained the following (in mm): 140 NaCl, 5.8 KCl, 1.3 CaCl_2_, 0.9 MgCl_2_, 0.7 NaH_2_PO_4_, 5.6 d-glucose, 10 HEPES-NaOH, pH 7.5.

Exocytosis was measured using the following intracellular solution (in mm): 106 Cs-glutamate, 20 CsCl, 3 MgCl_2_, 1 EGTA-CsOH, 5 Na_2_ATP, 0.3 Na_2_GTP, 5 HEPES-CsOH, 10 Na_2_-phosphocreatine, pH 7.3. Data acquisition was controlled by pClamp software (RRID:SCR_011323) using Digidata 1320A or 1440A boards (Molecular Devices). Recordings were low-pass filtered at 2.5 kHz (8-pole Bessel) and sampled at 5 kHz and stored on computer for off-line analysis (Origin: OriginLab, RRID:SCR_002815).

**Figure 1.**
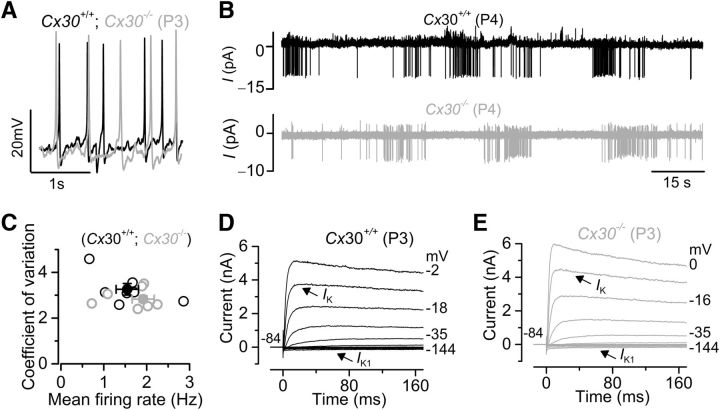
Connexins do not alter the biophysical properties of immature IHCs. ***A***, Spontaneous APs recorded from IHCs under whole-cell current-clamp configuration from P3 *Cx30(*−/−*)* mice and control littermates (+/+). In this and the following figures, black represents control (wild-type or heterozygous) and gray represents mutant or knock-out mice. ***B***, APs in cell-attached voltage clamp recorded from a P4 control (top) and P4 *Cx30(*−/−*)* (bottom) IHC. ***C***, Coefficient of variation from each IHC against their firing rate. Open symbols represent data from single IHCs. Closed symbols represent averages. ***D***, ***E***, Potassium currents elicited from P3 IHCs by applying depolarizing voltage steps in 10 mV nominal increments from −144 mV, starting from the holding potential of −84 mV. Recordings were performed at body temperature.

**Figure 2.**
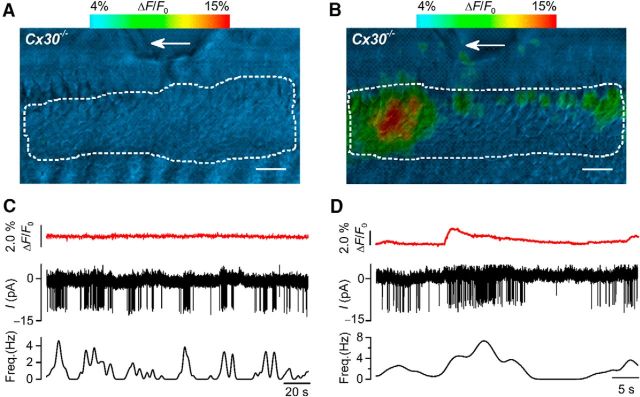
Spontaneous APs in IHCs are present in the absence of Ca^2+^ signals from nonsensory cells in *Cx30(*−/−*)* mice. ***A***, ***B***, Representative false-color images of Fluo-4 fluorescence changes (Δ*F*/*F*_0_), encoded as shown by the color scale bar (top) and obtained as maximal projection rendering of all frames recorded in 200 s (10 frame/s). The images show a small part of the GER in the proximity of the patched IHC (arrows) from P6 *Cx30(*−/−*)* mice. Note the absence (***A***) or some residual (***B***) Ca^2+^ signals from nonsensory cells in the GER. IHCs were patched from the pillar side to prevent damage to the GER. Scale bar, 10 μm. ***C***, ***D***, Simultaneous recording of Ca^2+^ transients in the nonsensory cells present in the GER using fluorescence imaging (Δ*F*/*F*_0_; see Materials and Methods) from white ROI delineated by the dashed white line in panel ***A*** and ***B***, respectively. Middle panels, IHC firing activity using cell-attached patch clamp. Bottom panels, Changes in AP frequency during the recordings. APs were present even when Ca^2+^ transients in the GER were absent (***A***; ***C***, top), but their frequency increases during the residual Ca^2+^ transients in *Cx30(*−/−*)* mice (***B***; ***D***, top). Recordings were performed at body temperature.

Membrane potentials were corrected for the voltage drop due to the series resistance *R*_s_ (2.7 ± 0.2 mΩ, *n* = 98) and liquid junction potential (K^+^- and Cs^+^-based intracellular solution: −4 mV and −11 mV, respectively). The Mini Analysis Program (RRID:SCR_002184: Synaptosoft) was used to detect spike events in cell-attached recordings. The AP frequency in Figure 1 was calculated as the reciprocal of the mean interspike interval for each cell and an indication of the spread of interspike interval values about the mean was obtained by calculating the coefficient of variation, equal to the SD divided by the mean. The firing rates in Figure 2 were estimated by convolving spike trains with a Gaussian kernel (SD 1 s) (Cunningham et al., 2009).

##### Statistical analysis.

Statistical comparisons of means were made by Student's two-tailed *t* test. Mean ± SEM values are reported; *p* < 0.05 indicates statistical significance.

##### Calcium dye loading in cochlear preparations.

For calcium dye loading, acutely dissected preparations were incubated for 40 min at 37°C in DMEM/F12, supplemented with fluo-4 AM (final concentration 16 μm; Thermo Fisher Scientific). The incubation medium contained also pluronic F-127 (0.1%, w/v, Sigma-Aldrich), and sulfinpyrazone (250 μm) to prevent dye sequestration and secretion. Preparations were then transferred to the microscope stage and perfused with extracellular solution for 20 min to allow for deesterification before initiating image acquisition.

##### Confocal Ca^2+^ imaging.

Ca^2+^ signals were recorded using a custom-built spinning disk confocal microscope (Ceriani et al., 2016a). Fluorescence excitation was produced by light emitted from a 470 nm LED (M470L2, Thorlabs) filtered through a BP460–480 filter (Olympus), and directed onto the sample through a 515 DCXR dichromatic mirror (Chroma Technology). Fluo-4 emission was filtered through a 535/43M bandpass interference filter (Edmund Optics). Confocal fluorescence images were formed by a water-immersion objective (40× NA 0.8, Olympus) and projected onto a scientific-grade camera (PCO Edge; PCO AG) controlled by software developed in the laboratory. Image sequences of Fluo-4 fluorescence were acquired continuously at 10 frames per second with 100 ms exposure time. To synchronize image acquisition and electrical recordings, we sampled the 5 V pulse that signals active exposure of the camera. Ca^2+^ signals were measured as relative changes of fluorescence emission intensity (Δ*F*/*F*_0_). Δ*F* = *F* − *F*_0_, where *F* is fluorescence at time *t* and *F*_0_ is the fluorescence at the onset of the recording; *F*_0_ was comparable among the five different cochlear preparations used for these experiments (see Results).

##### Transmission electron microscopy.

For transmission electron microscopy, cochleae dissected from 4-week-old mice were prepared as previously described (Mahendrasingam et al., 2011). Briefly, cochleae were fixed by perfusion with 2.5% glutaraldehyde in 0.1 m sodium cacodylate buffer containing 2 mm calcium chloride, pH 7.4, and immersed in the same fixative for 2 h. Cochleae were then washed in 0.1 m sodium cacodylate buffer containing 2 mm calcium chloride, pH 7.4, fixed in 1% osmium tetroxide in the same buffer for 1 h and decalcified in 5.5% EDTA/0.1% PFA solution for 3–4 d at 4°C. Decalcified cochleae were sectioned into 100-μm-thick slices, using a vibratome (Leica) equipped with a Gillette Platinum blade, and postfixed in uranyl acetate and in OsO_4_. After dehydration through a graded series of ethanol, the samples were cleared in propylene oxide, embedded in the Epoxy resin (Epon 812), and polymerized at 60°C for 72 h. From each embedded vibratome slice, 65 nm thin serial sections were cut in the region of interest using Leica EM UC7 ultramicrotome (Leica Microsystems) and collected on formvar-carbon coated slots. EM images of inner hair cells and ribbon synapses were acquired from thin serial sections using a FEI Tecnai-12 electron microscope (FEI) equipped with a VELETTA CCD digital camera (Soft Imaging Systems). Quantification of the number of synapse-associated vesicles was performed using the iTEM software (Soft Imaging Systems).

## Results

### AP activity in IHCs is normal in the absence of connexins 30 and 26

Spontaneous Ca^2+^-dependent AP activity occurs in IHCs of the mammalian cochlea during prehearing stages (Glowatzki and Fuchs, 2000; Beutner and Moser, 2001; Marcotti et al., 2003; Johnson et al., 2011, 2012). The frequency of these APs has been shown to be directly modulated not only by the efferent neurotransmitter ACh (Glowatzki and Fuchs, 2000), but also by the release of ATP from nonsensory cells in the GER (also known as Kölliker's organ) (Johnson et al., 2011). Recent studies have challenged this view and have proposed that AP activity in IHCs is not spontaneous but is instead triggered by Ca^2+^ waves (Tritsch et al., 2007, 2010; Wang et al., 2015) mediated by the release of ATP from connexin hemichannels (Anselmi et al., 2008; Majumder et al., 2010; Schütz et al., 2010; Rodriguez et al., 2012; Ceriani et al., 2016b). To address this controversial issue, we investigated AP activity in immature IHCs using connexin knock-out mice. To approach normal physiological recording conditions as best as possible, we performed experiments at body temperature (34°C–37°C), using 1.3 mm Ca^2+^ and 5.8 mm K^+^ in the extracellular solution, which mimics the perilymph surrounding the basolateral pole of hair cells (Wangemann and Schacht, 1996). For whole-cell recordings, 1 mm EGTA was used as the intracellular calcium buffer, which was previously assessed using perforated patch recordings where the mobile endogenous Ca^2+^ buffer is retained in the cell (Johnson et al., 2008).

We initially used *Cx30(*−/−*)* mice (Teubner et al., 2003) in which the mRNA and protein expression of Cx30 is abolished and, during prehearing stages, those of Cx26 are also reduced to only ∼10% of that present in wild-type mice (Ortolano et al., 2008; Boulay et al., 2013). In these *Cx30(*−/−*)* mice, the occurrence of spontaneous ATP-induced Ca^2+^ wave events in the GER is largely reduced (Rodriguez et al., 2012). Therefore, if the AP activity in IHCs were solely dependent on ATP-induced signaling from the nonsensory cells (Tritsch et al., 2007; Wang et al., 2015), it should be either absent or strongly reduced in these *Cx30(*−/−*)* mice. However, using either whole-cell current clamp (Fig. 1*A*) or cell-attached voltage clamp (Fig. 1*B*), we recorded spontaneous APs in IHCs from both wild-type and littermate P3-P4 *Cx30(*−/−*)* mice. In the cell-attached condition, APs take the form of capacitative currents that are abolished when IHCs are superfused with a Ca^2+^-free solution (Johnson et al., 2011). Long-lasting cell-attached recordings allowed us to investigate whether the firing pattern of APs in IHCs was affected by the genetic ablation of connexins in nonsensory cells. We used the coefficient of variation as a quantitative measure of regularity in spontaneous spike firing and found that in IHCs from wild-type (control) mice (3.26 ± 0.25, P4, *n* = 7) it was similar to that measured in cells from *Cx30(*−/−*)* littermates (2.83 ± 0.26, P4, *n* = 9. *p* = 0.3) (Fig. 1*C*). We also found that the resting membrane potentials and the size of immature K^+^ currents (Fig. 1*D* and Fig. 1*E*, respectively; Table 1) from control IHCs were similar to those of *Cx30(*−/−*)* littermates.

**Table 1.** Properties of immature and mature IHCs from Cx30 and Cx26 knock-out mice*^a^*

	Immature Cx30 KO(P3)		Adult Cx30 KO (P16-P18)		Cx26^Sox10-Cre^ (P25)		Cx30 Δ/Δ (P25)
	+/+	−/−	+/+	−/−	+/+	−/−	Δ/Δ
Resting potential (mV)	−57.7 ± 1.6 (4)	−58.5 ± 0.5 (4)	−74.4 ± 1.1 (11)	−69.3 ± 1.2 (11)	−69.2 ± 1.9 (3)	−73.5 ± 1.4 (8)	−73.5 ± 2.6 (5)
*I*_K1_ at −124 mV (pA)	−120 ± 31 (4)	−126 ± 25 (9)	—	−121 ± 13 (9)	—	?	—
*I*_K_ at 0 mV (nA)	4.5 ± 0.3 (5)	3.9 ± 0.2 (9)	12.8 ± 1.5 (10)	10.4 ± 0.8 (9)	13.1 ± 1.5 (3)	10.7 ± 1.0 (8)	13.5 ± 0.4 (6)
*I*_K,f_ at −25 mV (nA)	—	—	1.8 ± 0.3 (10)	—	3.7 ± 0.2 (3)	1.4 ± 0.2 (8)	2.8 ± 0.2 (6)
*I*_K,n_ at −124 mV (pA)	—	—	98 ± 67 (7)	—	302 ± 17 (3)	144 ± 17 (8)	349 ± 26 (5)

*^a^p* Data are mean ± SEM. Values in parentheses are number of hair cells. *I*_K1_, Inward rectifier K^+^ current (Marcotti et al., 1999); *I*_K_, delayed rectifier K^+^ current (Marcotti et al., 2003); *I*_K,n_, negatively activated K^+^ current carried by KCNQ4 channels (Marcotti et al., 2003); *I*_K,f_, Ca^2+^-activated K^+^ current (Kros et al., 1998); −, not present; ?, size of *I*_K1_ was difficult to quantify because, if present, it was masked by *I*_K,n_.

To confirm that Ca^2+^ waves are not required for AP activity in IHCs, we combined Ca^2+^ imaging from the GER (Majumder et al., 2010; Rodriguez et al., 2012) with cell-attached patch-clamp recordings from single IHCs (Johnson et al., 2011). Acutely dissected cochleae from *Cx30(*−/−*)* mice (*n* = 5) were loaded with the Ca^2+^ indicator Fluo-4 before starting electrophysiological recordings. We found that spontaneous AP activity was present in IHCs, even in the absence of detectable Ca^2+^ signals in the surrounding nonsensory cells (Fig. 2*A*,*C*). These results indicate that the origin of IHC electrical activity is independent from ATP-induced Ca^2+^ waves from nonsensory cells. In recordings where some Ca^2+^ signals remained in the nonsensory cells of the GER of *Cx30(*−/−*)* mice, likely due to the residual expression of Cx26, the intrinsic AP activity of the patched IHC showed a rapid and transient increase in its firing frequency, which was correlated with the Ca^2+^ wave (Fig. 2*B*,*D*). Also, at the beginning of the recordings in Figure 2*B*, *D*, the IHC fired APs, even in the absence of Ca^2+^ signals from nonsensory cells. This adds further support to the above findings showing that AP activity is intrinsic to IHCs and is extracellularly modulated by ATP-dependent Ca^2+^ waves in cochlear nonsensory cells.

### IHCs from *Cx30(*−/−*)* mice fail to acquire adult-type membrane currents

**Figure 3.**
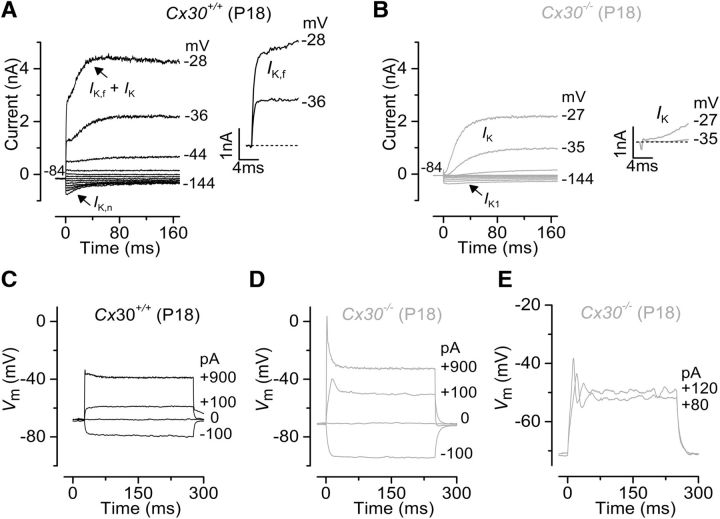
Current and voltage responses recorded from IHCs of *Cx30(*−/−*)* mice. ***A***, ***B***, Potassium currents recorded from P18 IHCs of wild-type (***A***) and littermate *Cx30(*−/−*)* (***B***) mice using depolarizing voltage steps in 10 mV nominal increments from the holding potential of −84 mV to the various test potentials shown by some of the traces. The adult-type currents (*I*_K,f_ and *I*_K,n_) were only present in IHCs from wild-type mice (***A***). IHCs from *Cx30(*−/−*)* mice retained the currents characteristic of immature cells (*I*_K,s_ and *I*_K1_). The presence of the rapidly activating *I*_K,f_ in control IHCs is evident when comparing the activation time course of the total outward currents shown in the insets on an expanded time scale. ***C–E***, Voltage responses elicited by applying hyperpolarizing and depolarizing current injections to control (***C***) and *Cx30(*−/−*)* adult IHCs (***D***, ***E***) from their respective membrane potentials. In some IHCs, depolarizing current injections caused slow APs at the onset of responses. Recordings were performed at room temperature.

Because genetic ablation of connexins from the cochlear sensory epithelium does not alter the basolateral membrane properties of IHCs during the first postnatal week (Fig. 1; Table 1), we set out to determine whether it impacts on the progression of IHC development. To address this question, we measured the biophysical properties of posthearing IHCs. Hair cell membrane capacitance (*C*_m_), which gives an estimate of the cell's surface area, showed that control IHCs (10.40 ± 0.25 pF, *n* = 14, P18-P25) where significantly bigger than those from *Cx30(*−/−*)* littermates (9.06 ± 0.30 pF, *n* = 17, P18–P24, *p* < 0.005), indicating that the absence of Cx30 impaired the normal growth of IHCs. We also recorded the total outward K^+^ current in mature IHCs by applying a series of depolarizing voltage steps in 10 mV increments from −144 mV (holding potential was −84 mV). The mature-type currents *I*_K,f_ (Kros et al., 1998) and *I*_K,n_ (Marcotti et al., 2003) were both present in P16-P18 control IHCs (Fig. 3*A*), but undetectable in cells from aged-matched *Cx30(*−/−*)* littermates (Fig. 3*B*; Table 1). In the latter, IHCs also retained the inward rectifier K^+^ current *I*_K1_, which is characteristic of an immature electrophysiological phenotype (Marcotti et al., 1999). Consistent with this conclusion, IHCs from *Cx30(*−/−*)* mice had a resting membrane potential (*V*_m_) significantly more depolarized than that measured in cells from control littermates (*p* < 0.01; Table 1), responded to depolarizing current injections with much larger voltage responses (Fig. 3*C*,*D*) and retained some ability to generate slow Ca^2+^ APs at the response onset (Fig. 3*E*).

**Figure 4.**
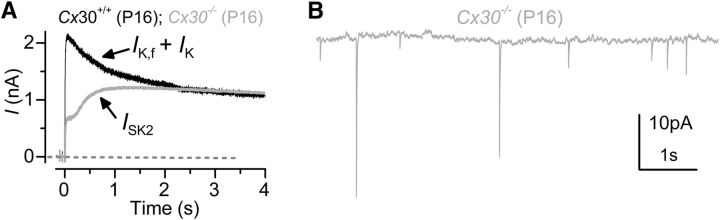
Efferent activity is still present in mature *Cx30(*−/−*)* IHCs. ***A***, Outward currents obtained by using a 4 s depolarizing step to 0 mV from the holding potential of −84 mV in control and *Cx30(*−/−*)* P16 IHCs (Marcotti et al., 2004). Although the SK2 current (*I*_SK2_) is normally downregulated after the onset of hearing (Glowatzki and Fuchs, 2000), it was still expressed in mature *Cx30(*−/−*)* IHCs. ***B***, Spontaneous IPSCs recorded from a P16 *Cx30(*−/−*)* IHC indicate that these IHCs retain the efferent endings that normally make only transient axosomatic synaptic contacts with IHCs during immature stages (Simmons et al., 1996; Katz et al., 2004). Recordings were performed at body temperature.

We also found that IHCs from mature *Cx30(*−/−*)* mice expressed SK2 channels (Fig. 4*A*) and showed IPSCs (Fig. 4*B*), which are driven by spontaneous release of ACh from the efferent fibers. This ACh-activated current, which is mediated by Ca^2+^ entering hair cells through a9a10-nAChRs and activates SK2 channels, is normally only present in immature IHCs (Glowatzki and Fuchs, 2000; Marcotti et al., 2004), at a time when efferent endings make transient axosomatic synaptic contact with these cells (Pujol et al., 1998). These findings further support the evidence that IHCs from *Cx30(*−/−*)* mice retain an immature phenotype.

### The biophysical properties of ribbon synapses and vesicle pool size is affected in IHCs from *Cx30(*−/−*)* mice

**Figure 5.**
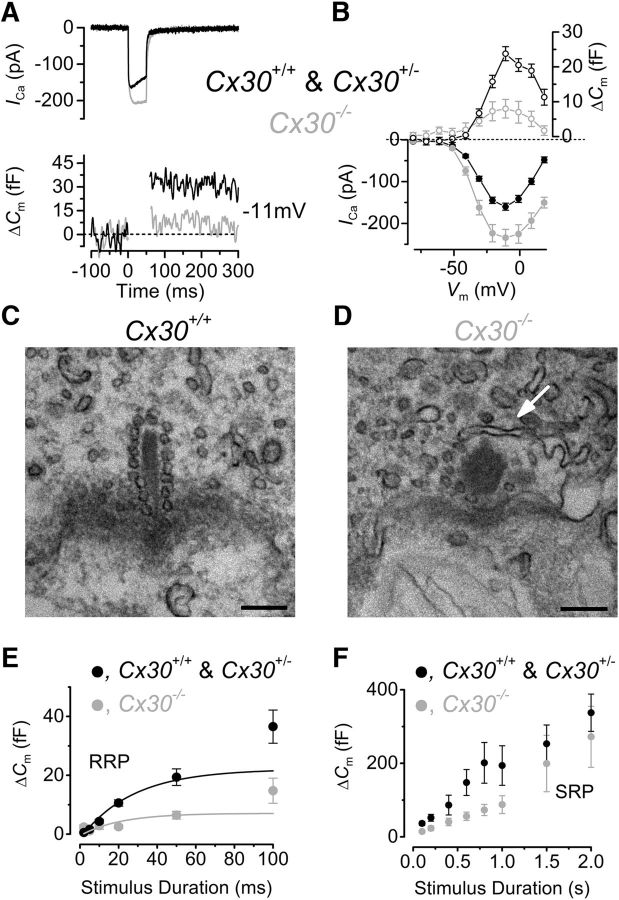
Exocytosis and ribbon morphology in *Cx30(*−/−*)* IHCs. ***A***, *I*_Ca_ and corresponding Δ*C*_m_ recorded from adult control and *Cx30(*−/−*)* IHCs obtained in response to 50 ms voltage steps, in 10 mV increments, from −81 mV. For clarity, only maximal responses at −11 mV are shown. ***B***, Average peak *I*_Ca_ (bottom) and Δ*C*_m_ (top) curve from control (P17–P25, *n* = 15) and *Cx30(*−/−*)* (P18–P24, *n* = 5) IHCs. ***C***, ***D***, Typical cross-sectional profiles of synaptic ribbons obtained from a control (***C***) and a *Cx30(*−/−*)* (***D***) IHC. Some of the synaptic vesicles are missing around the ribbon of the *Cx30(*−/−*)* IHC (arrow). Scale bar, 200 nm. ***E***, ***F***, Average Δ*C*_m_ from 12 control and 14 *Cx30(*−/−*)* IHCs in response to voltage steps from 2 ms to 2 s (to ∼−11 mV) showing the RRP (***E***) and SRP (***F***). ***E***, The points at 100 ms represent the recruitment of the SRP. Recordings were performed at body temperature.

We then tested whether the absence of connexins in nonsensory cells also affected ribbon synapse function in IHCs, which is crucial for sound encoding. Exocytosis was estimated by measuring increases in cell membrane capacitance (Δ*C*_m_) following 50 ms depolarizing voltage steps. We found that, in IHCs from adult *Cx30(*−/−*)* mice, the maximal size of the Ca^2+^ current (*I*_Ca_) was significantly larger (*p* < 0.001) than that of control cells (Fig. 5*A*,*B*; Control IHCs 159.9 ± 8.4 pA, *n* = 15, P17-P25; *Cx30(*−/−*)* IHCs 234.1 ± 19.8 pA, *n* = 5, P18-P24), but similar to that normally measured in immature IHCs from wild-type mice (Johnson et al., 2010). Despite the larger *I*_Ca_, the corresponding Δ*C*_m_ was significantly reduced in *Cx30(*−/−*)* mice (Fig. 5*A*,*B*; Control IHCs 23.8 ± 2.0 pF, *n* = 15; *Cx30(*−/−*)* IHCs 8.0 ± 2.7 pF, *n* = 5, *p* < 0.001), which is an indication of a reduced number of synaptic vesicles fusing to the plasma membrane. As such, the Ca^2+^ efficiency of exocytosis, which was measured by normalizing Δ*C*_m_ to the peak *I*_Ca_ (fF pA^−1^) was found to be significantly reduced in *Cx30(*−/−*)* IHCs (0.034 ± 0.022, *n* = 5) compared with control cells (0.150 ± 0.046, *n* = 15, *p* < 0.0001), but similar to that obtained in prehearing IHCs (0.053 ± 0.011, *n* = 7, P7). The reduced exocytosis in *Cx30(*−/−*)* IHCs was confirmed by transmission electron microscopy, which showed significantly (*p* < 0.001) fewer vesicles tethered to the ribbon synapses of IHCs from *Cx30(*−/−*)* mice (7 ± 1, *n* = 10; Fig. 5*D*) compared with control cells (13 ± 1, *n* = 11; Fig. 5*C*). Moreover, the synaptic ribbons of control IHCs exhibited the characteristic ellipsoidal morphology previously described in mature mouse IHCs (Sobkowicz et al., 1982), whereas those in cells from *Cx30(*−/−*)* mice retained the more spherical shape typical of immature synapses (Sobkowicz et al., 1982).

We also investigated the two major components of the releasable vesicle pools (Fig. 5*E*,*F*) by measuring Δ*C*_m_ in response to depolarizing voltage steps to −11 mV of increasing duration (from 2 ms to 2 s). As previously shown, short stimuli (usually up to 50 ms), applied to mouse IHCs in 1.3 mm extracellular Ca^2+^ and, at body temperature, reveal the number of vesicles docked at the active zones (readily releasable pool: RRP), whereas longer steps induce the release of vesicles from a secondarily releasable pool (SRP) (Johnson et al., 2010, 2013). We found that the size of the RRP in control IHCs (22.1 ± 2.7 fF, *n* = 12) was significantly larger than that obtained from *Cx30(*−/−*)* cells (7.1 ± 1.2 fF, *n* = 14, *p* < 0.0001; Fig. 5*E*), in agreement with the above findings (Fig. 5*A*,*B*). However, the initial release rate of the RRP was similar between the two genotypes (control IHCs: 898 ± 96 fF/s or 24275 ± 2594 vesicles/s, *n* = 12; *Cx30(*−/−*)* IHCs: 1437 ± 653 fF/s or 38840 ± 17646 vesicles/s, *n* = 14, values obtained from fits to individual IHCs), indicating that only the number of available vesicles, not their biophysics of release, was affected in *Cx30(*−/−*)* IHCs. In contrast, Δ*C*_m_ responses induced by long-lasting voltage steps (200 ms to 2 s) were found to be not significantly different (two-way ANOVA followed by the Bonferroni post test) between control and *Cx30(*−/−*)* IHCs (Fig. 5*F*), showing that the number of vesicles located further away from the active zones was not significantly affected by the absence of connexins in the nonsensory cells of the cochlear sensory epithelium.

Overall, these results indicate that IHCs from *Cx30(*−/−*)* mice, in which the protein expression of Cx26 is also largely downregulated, retain a prehearing phenotype and, as such, would be unable to encode correctly incoming sound stimuli.

### Deletion of Cx30, but preservation of Cx26 expression in Cx30^Δ/Δ^ mice, is sufficient for near-normal IHC development

**Figure 6.**
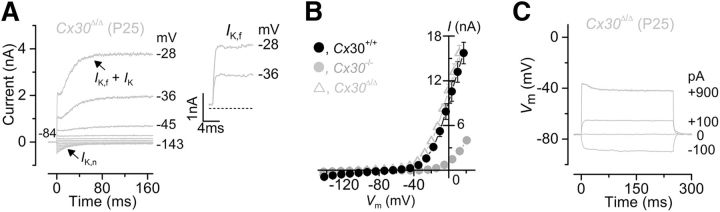
IHCs from *Cx30*^Δ/Δ^ knock-out mice develop normally. ***A***, Potassium currents recorded from mature IHCs (P25) of *Cx30*^Δ/Δ^ knock-out mice using the same voltage protocol described in Figure 3. Inset, The presence of *I*_K,f_ is evident. ***B***, Current-voltage curves measured from IHCs at 2 ms from the stimulus onset of *Cx30*^Δ/Δ^ (*n* = 6, P25), wild-type (*Cx30*^+/+^: *n* = 10, P16–P18), and *Cx30(*−/−*)* (*Cx30*^−/−^: *n* = 9, P16–P18) mice. ***C***, Voltage responses recorded from a mature IHC of a *Cx30*^Δ/Δ^ mouse, which were elicited by applying hyperpolarizing and depolarizing current injections. Recordings were performed at room temperature.

**Figure 7.**
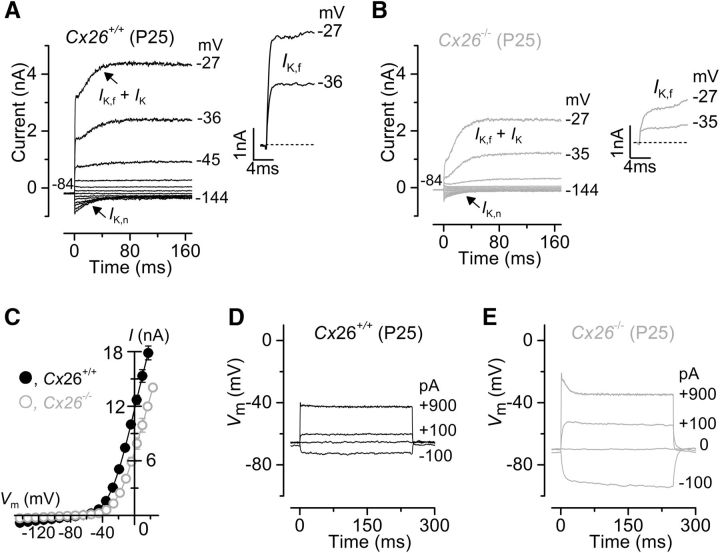
Current and voltage responses recorded from IHCs of *Cx26^Sox10-Cre^* mice. ***A***, ***B***, Potassium currents recorded from P25 IHCs of wild-type (***A***) and littermate *Cx26^Sox10-Cre^* (***B***) mice using the same voltage protocol as described in Figure 3. Both the rapidly activating *I*_K,f_ and the negatively activated *I*_K,n_ were present in the *Cx26^Sox10-Cre^* IHC, although reduced in size compared with the control cell. ***C***, Current-voltage curves obtained as in Figure 6*B* from IHCs of wild-type (*Cx26*^+/+^: *n* = 3, P25) and *Cx26^Sox10-Cre^* (*Cx26*^−/−^: *n* = 8, P25) mice. ***D***, ***E***, Voltage responses elicited as described in Figure 3 from control (***D***) and knock-out (***E***) adult IHCs from the same cells shown in ***A*** and ***B***, respectively. Recordings were performed at room temperature.

Recent findings have shown that deletion of *Cx30* does not cause any measurable hearing loss in young adults of the *Cx30*^Δ/Δ^ mouse model. In these mice, Cx30 is absent, as in the *Cx30(*−/−*)* mice, but they retain a higher expression level of Cx26 (∼50%) (Boulay et al., 2013) compared with the *Cx30(*−/−*)* (∼10%) (Teubner et al., 2003). Therefore, the prediction was that the biophysical properties of IHCs from *Cx30*^Δ/Δ^ mice should be indistinguishable from those of wild-type control cells. Indeed, we found that adult (P25) IHCs from *Cx30*^Δ/Δ^ mice expressed both *I*_K,f_ and *I*_K,n_ (Fig. 6*A*) with peak currents matching that of control cells and, as such, much larger than that of *Cx30(*−/−*)* mice (Fig. 6*B*; see also Table 1). Voltage responses (Fig. 6*C*) and resting membrane potentials (Table 1) of *Cx30*^Δ/Δ^ IHCs were also similar to those measured in control cells (Fig. 3*C*). A possible conclusion is that the near-complete absence of Cx26 is likely to be responsible for the IHC defects observed in *Cx30(*−/−*)* mice (Figs. 3; 5). We tested this hypothesis by performing recordings from adult IHCs of *Cx26^Sox10-Cre^* mice (*Cx26^loxp/loxp^* × *Sox10-Cre*; see Materials and Methods; Fig. 7), in which Cx26 is not present in the sensory epithelium of the cochlea, whereas the normal expression of Cx30 is delayed, such that it only starts to be present in the GER during the second postnatal week and becomes normal from approximately P14 (Crispino et al., 2011). Adult IHCs from *Cx26^Sox10-Cre^* mice (P25) showed a current profile (Fig. 7*B*,*C*) similar to that of normal cells (Fig. 7*A*,*C*) but with a significantly reduced size of both *I*_K,f_ (*p* < 0.0001) and *I*_K,n_ (*p* < 0.001) (Table 1). Voltage responses (Fig. 7*D*,*E*) were different between control and *Cx26^Sox10-Cre^* mature IHCs, reflecting the reduced expression of *I*_K,f_ and *I*_K,n_. Therefore, the expression of Cx30 in *Cx26^Sox10-Cre^* mice during the second postnatal week, although reduced, is sufficient to trigger a partial maturation of the IHCs, indicating that both connexins contribute to the acquisition of normal hearing.

## Discussion

Using near-physiological recording conditions, we have shown that, different from previous suggestions (Tritsch et al., 2007, 2010), ATP released from nonsensory cells of the immature mouse cochlea is not required for the generation of APs in IHCs. Our data from *Cx30(*−/−*)* mice show that immature IHCs exhibit spontaneous AP activity that is similar to that recorded in control cells and, as such, is independent from Cx26/Cx30 gap-junction channels or hemichannels. However, the presence of ATP-induced signaling in nonsensory cells increases the AP frequency in IHCs. We also found that a threshold level for the expression of both Cx26 and Cx30 is required for the acquisition of the mature sensory profile in IHCs and, as such, for the normal hearing.

### Contribution of Cx26 and Cx30 to the hearing phenotype

Cx26 and Cx30 are the predominant connexins in the mammalian cochlea and the only isoforms expressed in the sensory epithelium (Jagger and Forge, 2015; Wingard and Zhao, 2015). These connexins have been shown to form extrajunctional hemichannels (Anselmi et al., 2008; Majumder et al., 2010) and also to coassemble to form homomeric or heteromeric gap junction channels between nonsensory cells (Forge et al., 2003; Martínez et al., 2009). This cell syncytium does not include the sensory IHCs or OHCs (e.g., Oesterle and Dallos, 1990; Jagger and Forge, 2006; Majumder et al., 2010), yet connexins have been shown to be crucial for hearing as deletion of Cx26 (e.g., Cohen-Salmon et al., 2002; Crispino et al., 2011) and mutation of Cx26 or Cx30 cause hearing loss in mice (Schütz et al., 2010, 2011) and humans (Wang et al., 2011; e.g., del Castillo and del Castillo, 2012). Deletion of Cx30 is normally associated with substantial downregulation of Cx26 expression in *Cx30(*−/−*)* mice (Ortolano et al., 2008) as well as in DFNB1 patients (Lerer et al., 2001; Pallares-Ruiz et al., 2002; del Castillo et al., 2002; Rodriguez-Paris and Schrijver, 2009). The highly reduced expression of Cx26 in *Cx30(*−/−*)* mice occurs because the two genes expressing these proteins are only 50 kb apart, such that large deletions in the DFNB1 locus in humans or large insertions (*lacZ* or *neo* cassette) in mice cause downregulation in the expression of both Cx26 and Cx30 (Boulay et al., 2013). Overexpression of Cx30 by viral transduction of *Cx30(*−/−*)* organotypic cultures restored Cx26 mRNA expression at levels similar to those in wild-type controls (Ortolano et al., 2008). On the other hand, viral transduction of *Cx26^loxP/loxP^* cultures with a vector encoding the bacterial Cre recombinase reduced mRNA levels of both connexins (Ortolano et al., 2008). Normal hearing was restored in *Cx30(*−/−*)* when Cx26 was overexpressed (Ahmad et al., 2007), and preserved in *Cx30*^Δ/Δ^ mice where approximately half of the normal Cx26 was retained (Boulay et al., 2013). Together, these results indicate that the expression level of both connexin genes is closely interrelated.

Our results show that an absence of Cx30 in *Cx30*^Δ/Δ^ mice (with ∼50% of the normal Cx26), does not prevent the normal functional maturation of prehearing IHCs. By contrast, in *Cx30(*−/−*)* mice, which have no Cx30 and only ∼10% of residual Cx26, IHCs failed to achieve functional maturation and retained their prehearing biophysical and morphological configuration. In *Cx26^Sox10-Cre^* mice, in which Cx26 is not expressed in the sensory epithelium (Crispino et al., 2011), IHCs were able to develop, but only partially, indicating that the residual Cx30 expression in these mice was sufficient to promote some initial maturation. Therefore, IHC functional differentiation into mature sensory receptor is initiated provided that the expression of either connexin reaches a threshold level, which is likely to be ∼50% for Cx26 (as in *Cx30*^Δ/Δ^ mice). For Cx30 this level is not known, but because IHC maturation was initiated in *Cx26^Sox10-Cre^* mice, the threshold could be similar to that of Cx26. This functional interaction between Cx26 and Cx30, recently demonstrated in mouse models (Ortolano et al., 2008), has also been hypothesized in humans based on observations using super-resolution microscopy (Liu et al., 2016).

In mature *Cx30(*−/−*)* mice, cochlear hair cells begin to degenerate and by 4 months of age cell loss is extensive (Teubner et al., 2003). This progressive degeneration has been previously linked to a complete absence of endocochlear potential in these *Cx30(*−/−*)* mice (Teubner et al., 2003). However, a similar pattern of hair cell degeneration has been described in Cx26^Sox10-Cre^ mice that show a residual (40 mV) endocochlear potential (Crispino et al., 2011) and *Beethoven* mice that have a normal endocochlear potential (Marcotti et al., 2006). Therefore, the progressive loss of hair cells in *Cx30(*−/−*)* and *Cx26^Sox10-Cre^* mice is likely to be a direct consequence of their inability to express adult-like currents, such as that carried by KCNQ4 channel (*I*_K,n_) (Kharkovets et al., 2006; Marcotti et al., 2006). Mutation in KCNQ4 channels has been shown to lead to progressive hearing loss in DFNA2 patients (Kubisch et al., 1999), suggesting that *I*_K,n_ may be important for maintaining hair cell viability. The presence of immature IHCs and the reduced or absent endocochlear potential will, together, lead to hearing loss in mice and humans with mutations in connexins.

### Mechanisms underlying the interaction of cochlear nonsensory cells with IHCs

The exact physiological role of connexin channels in nonsensory cells of the mammalian cochlea is still unclear. Current evidence indicates that Cx26 and Cx30 are crucial for maintaining the ionic and metabolic homeostasis of the inner ear and mediating intercellular signaling in the nonsensory epithelium (Mammano, 2013; Jagger and Forge, 2015; Wingard and Zhao, 2015). We now show that these connexins are also involved in the maturation of the biophysical and morphological properties of IHCs, which normally occurs at around the onset of hearing. It is known that Cx26 and Cx30 hemichannels in the nonsensory cells of the cochlea release ATP under physiological conditions (Anselmi et al., 2008; Rodriguez et al., 2012). The binding of extracellular ATP to G-protein coupled P2Y receptors on nonsensory cells activates the phospholipase-C dependent generation of IP_3_, which then binds to its receptors on the ER and promotes Ca^2+^ release, raising the cytosolic free [Ca^2+^] and additional release of ATP (Beltramello et al., 2005; Piazza et al., 2007). This cascade of events enables the propagation of Ca^2+^ signals as regenerative and coordinated intercellular Ca^2+^ waves in cochlear nonsensory cells, sustained by ATP-induced ATP release (Beltramello et al., 2005; Piazza et al., 2007; Rodriguez et al., 2012; Ceriani et al., 2016b). Recent work has proposed that this ATP-dependent Ca^2+^ signaling is required to generate AP activity in IHCs (Tritsch et al., 2007, 2010; Wang et al., 2015). However, we now show that, in early postnatal *Cx30(*−/−*)* mice, which have a greatly reduced frequency of ATP-dependent Ca^2+^ activity in the nonsensory cells of the GER (Rodriguez et al., 2012), IHCs retain the ability to fire spontaneous APs (Fig. 1). However, the remaining Ca^2+^ waves originating in the nonsensory cells of *Cx30(*−/−*)* mice were able to increase the IHC firing activity (Fig. 2), supporting previous data showing that ATP released from nonsensory cells has mainly a modulatory role on APs in IHCs (Johnson et al., 2011). Despite the presence of AP activity, IHCs from *Cx30(*−/−*)* mice do not properly mature into sensory receptors.

A comparable failure in IHC maturation to that of *Cx30(*−/−*)* mice has previously been described in mutant or transgenic mice showing defects in the mechanoelectrical transducer current (*tmc1*: Marcotti et al., 2006; Kawashima et al., 2011; *Eps8*: Zampini et al., 2011; *Myo6*: Roux et al., 2009), which is believed to be directly implicated in the persistence of spontaneous AP activity in IHCs *in vivo* during the second postnatal week (Johnson et al., 2012). AP activity over this time window, just before the onset of hearing at P12, is used by IHCs to promote their own functional maturation *in vivo* (Johnson et al., 2013). Because connexin-dependent ATP-induced Ca^2+^ signaling activity from the GER can modulate the frequency of APs in immature IHCs (Fig. 2), it is conceivable that the reduced ATP release from nonsensory cells in *Cx26^Sox10-Cre^* and *Cx30(*−/−*)* mice is likely to alter the normal pattern of spontaneous AP activity in IHCs during the second postnatal week and, as such, IHC maturation. This is also supported by the evidence that the appearance of Cx30 during the second postnatal week in *Cx26^Sox10-Cre^* mice, in which Cx26 is absent, is sufficient to promote a partial maturation of IHCs. The release of ATP from nonsensory cells could either affect the IHC firing activity directly, via the reduced activation of P2 receptors present in these cells (Housley et al., 2006; Tritsch et al., 2007; Johnson et al., 2011), or indirectly via the elevation of K^+^ around the IHCs following the activation of ATP autoreceptors in neighboring nonsensory cells (Wang et al., 2015).
